# Arterial blood architecture of the maxillary sinus in dentate specimens

**DOI:** 10.3325/cmj.2013.54.180

**Published:** 2013-04

**Authors:** Lumnije Kqiku, Robert Biblekaj, Andreas H. Weiglein, Xhylsime Kqiku, Peter Städtler

**Affiliations:** 1 Division of Preventive and Operative Dentistry, Endodontics, Pedodontics, and Minimally Invasive Dentistry, Department of Dentistry and Maxillofacial Surgery, Medical University, Graz, Austria; 2 Institute of Anatomy, Medical University, Graz, Austria; 3 Department of Internal Medicine, Division of Pulmonology, Medical University, Graz, Austria

## Abstract

**Aim:**

To describe vascular anatomy of the maxillary sinus in dentate specimens dissected from human cadavers.

**Methods:**

Twenty dentate maxillary specimens were dissected, anatomically prepared, and injected with liquid latex for a better visualization of the maxillary sinus artery.

**Results:**

We found an intraosseous anastomosis in 100% and an extraosseous anastomosis in 90% of the cases. The anterior lateral wall of the maxillary sinus was transversed by two anastomoses between the posterior superior alveolar artery (PSAA) and the infraorbital artery (IOA). The PSAA was divided into a gingival and dental branch. The gingival branch anastomosed with the terminal extraosseous branch of the extraosseous anastomosis (EOA) and the dental branch with the intraosseous branch of the intraosseous anastomosis (IOA). The mean distances from the alveolar ridge to the extraosseus anastomosis were 16 mm for the second maxillary molar, 12.3 mm for the first maxillary molar, and 13.1 mm for the second maxillary premolar. The mean distances from the intraosseous anastomosis to the alveolar ridge were 17.7 mm for the second maxillary molar, 14.5 mm for the first maxillary molar, and 14.66 mm for the second maxillary premolar.

**Conclusion:**

These findings provide relevant data for clinical dentistry in order to avoid bleeding complications and minimize the risk of injury to the arterial network of the maxillary sinus during surgical procedures in the dentate maxilla region.

The blood supply of the maxillary sinus is derived from following branches of the maxillary artery (MA): the posterior superior alveolar artery, the infraorbital artery, and the descending palatine artery (1-5). The deepest point of the maxillary sinus is usually located in the region of the molar roots from the first and second molar, the two most commonly dehiscent teeth in the maxillary sinus (6). In order to prevent bleeding complications and maxillary bone necrosis during surgical procedures and oral operative treatments involving this region (osteotomy, endodontic surgery, dental implant, treatment of injuries), it is very important to have anatomical knowledge of maxillary sinus vascularization, especially the distribution and the distance from the alveolar ridge in the clinically relevant level of maxillary teeth. Anatomy and topography of the maxillary sinus artery have been frequently described and the most recent studies investigated the maxillary sinus artery in the atrophic maxillas (7-11). However, the vertical distances in dentate specimens from the alveolar ridge to the extraosseous and intraosseous anastomosis at the level of the second maxillary premolar and molar teeth have not been completely described yet. Therefore, the aim of this study was to describe the maxillary sinus vascularization at a glance, the anastomoses, and the distances from the alveolar ridge to the extraosseous and intraosseous anastomosis in the specific sectors via direct observation in dentate dissected human cadaver specimens.

## Materials and methods

**Figure 1:**
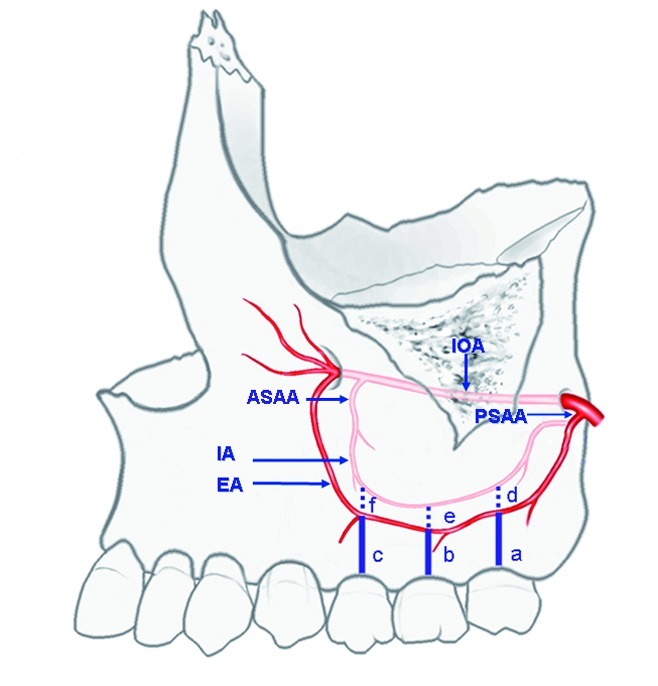
Selected sectors for anatomical investigation/distance measured from intraosseous anastomosis (IA) and extraosseous anastomosis (EA) to alveolar ridge in specific levels of the maxillary teeth graded as follows: **(A)** Distance from the alveolar crest at the level of the second maxillary molar to the IA. **(B)** Distance from the alveolar crest at the level of the first maxillary molar to the IA. **(C)** Distance from the alveolar crest at the level of the second maxillary premolar to the IA. **(D)** Distance from the alveolar crest at the level of the second maxillary molar to the EA. **(E)** Distance from the alveolar crest at the level of the first maxillary molar to the EA. **(F)** Distance from the alveolar crest at the level of the first maxillary premolar to the EA.

We dissected 20 completely dentate maxillary segments (10 from the right and 10 from the left side) of 5 male and 5 female human cadavers aged 46-94 years (mean ± standard deviation, 73 ± 13.6) at the Institute of Anatomy of the Medical University of Graz in 2011. The cadavers were fixed by Thiel’s method (12) and the external carotid arteries were injected with liquid latex (the Thiel’s DGM 85 substance/masses for arterial injection) consisting of dextrin, latex, and lead tetroxide (red lead) for precise and easy identification of the arteries (13). After fixation with formalin the skulls were divided sagitally into halves with an electric band saw. The mandible was separated and removed. One half of each maxilla was prepared using a scalpel and forceps. The soft tissues were carefully removed using instruments for microsurgery and all maxillary segments were separated. In order to access the maxillary sinus, the nasal conchae were removed and the lateral wall of the nasal cavity was fenestrated. Hence, the third portion (pterygopalatine portion) of maxillary artery (MA), and the posterolateral and antral wall of the maxillary sinus were exposed to analyze the anatomy of the superior alveolar artery (PSAA) and the infraorbital artery (IOA), the anastomoses, the number of branches, and the distance from the deepest caudal point of the extraosseous and intraosseous anastomoses of the PSAA and IOA to the alveolar ridge at the level of the second premolar and of the first and the second maxillary molar (Figure 1). All dissections were documented by line drawings and a digital camera (Sony Cybershot 7.2 MP, Sony Austria GMBH, Vienna, Austria).

## Results

**Figure 2:**
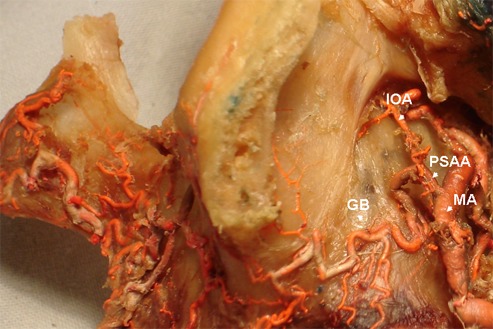
Gingival branch (extraosseous terminal branch) of the posterior superior alveolar artery (PSAA).

**Figure 3:**
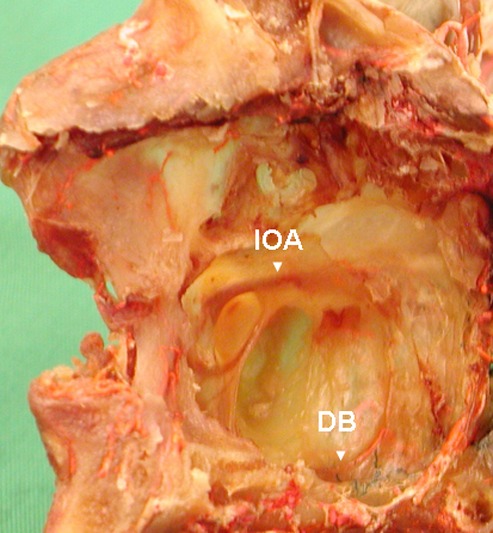
Dental branch (intraosseous terminal branch) of the posterior superior alveolar artery (PSAA).

**Figure 4:**
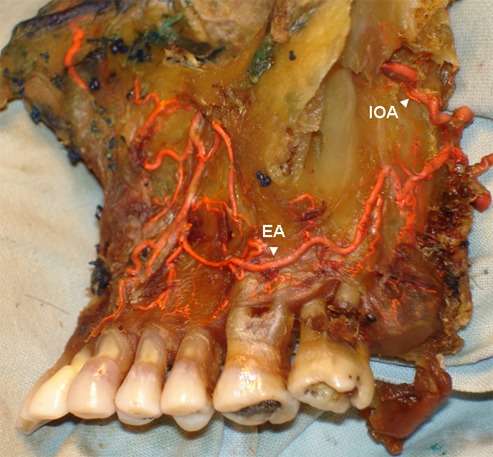
Maxilla prepared for anatomical study (vestibular view). Extraosseous anastomosis (EA).

**Figure 5:**
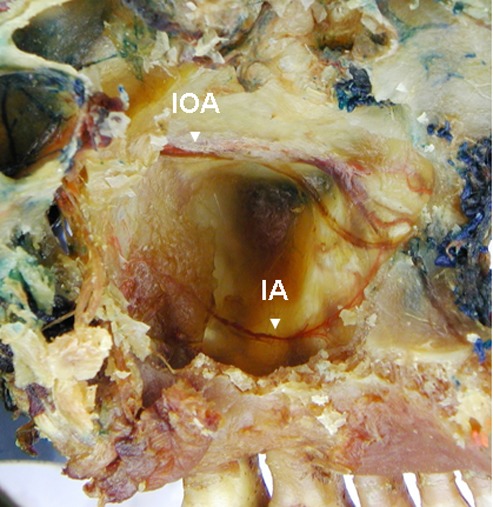
Maxilla prepared for anatomical study (oral view). Intraosseous anastomosis (IA).

In 12 of the 20 specimens (60%), the PSAA and the IOA had a common trunk from the MA, whereas in 8 specimens (40%) the PSAA and the IOA branched separately from the MA. The PSAA was located caudally in close contact with the bone and periosteum of the maxillary tuberosity and was divided into a gingival (extraosseous terminal branch) (Figure 2) and a dental branch (intraosseous terminal branch) (Figure 3). The gingival branch anastomosed in 18 specimens (90%) with the extraosseous terminal branch of the IOA and formed the arterial arcade named extraosseous anastomosis (EA) (Figure 1 and Figure 4). The dental branch of the PSAA anastomosed with the intraosseous branch (anterior superior alveolar artery/ASAA) of the IOA and formed the arterial arcade named intraosseous anastomosis (IA) (Figure 1 and Figure 5). This anastomosis was found in all specimens. The mean distances from the alveolar ridge in specific sectors of molar teeth to the caudal point of the EA were 16 mm for the second maxillary molar, 12.3 mm for the first maxillary molar, and 13.1 mm for the second maxillary premolar. The mean distances from IA to the alveolar ridge were 17.7 mm for the second maxillary molar, 14.5 mm for the first maxillary molar, and 14.7 mm for the second maxillary premolar.

## Discussion

In this study, the anastomoses of the gingival and the dental branches, as well as their relationship/distance to the alveolar ridge in dentate specimens were analyzed in predefined clinically relevant levels, in particular at the first and the second molar and at the second maxillary premolar, as these parameters have still not been completely described. The anatomical dissection methods used for assessment of the anatomical structure are the most commonly used methods of investigation. This anatomical study exerted macroscopic dissection to describe the arterial blood supply of the maxillary sinus. In anatomical studies by Solar et al (7) and Traxler et al (8), the EA was found in 44% and 33% of cases, respectively, and showed a distance of 22.8-26 mm from the alveolar crest. However, in our study the EA was found in 90% of cases and the distance to the alveolar ridge was smaller for all specific sectors of the selected maxillary teeth (12.3-16 mm). The IA was found in 100% of cases, which was also shown in other two studies (8,9) but not in the study of Rodella et al (10), who found the IA only in 66% of cases. The mean distance from the IA to the alveolar crest was found to be 18.9-19.6 mm (7,8), whereas in our study this distance was from 14.66-17.72 mm. This is in agreement with the results by Elian et al (11) and Mardinger et al (14), who found the distances of 16.4 mm (11) and 16.9 mm (14), respectively. Kim et al (15) showed that the distance from the PSAA to the alveolar crest was greater in the premolar (18.90 ± 4.21 mm) than in the molar area (15.45 ± 4.04 mm). Generally, the present study showed that the both anastomoses were located slightly lower as than in other studies (7,8,15). The discrepancy from the results of Solar et al (7), Traxler et al (8), and Kim et al (15) may be due to differences in the investigated specimens as we only used dentate maxillae segments. The presence of teeth plays a relevant role in determining the location of the vessel. Rosano et al (16) showed that edentulous sextants in the posterior maxilla showed a lower height and smaller width of the ridge than contralateral dentate sextants (17). Recently, it has been reported that the average height of intraosseous artery was 13 ± 3.2 mm in the distal doors and 18 ± 6.1 mm in the mesial doors (10). They concluded that the risk of vascular damage in sinus floor elevation surgery was a real problem for the oral surgeon. This is in accordance with our results, with the only difference from other studies being that the distances were not measured at two levels from the distal and mesial doors but in the three relevant specific sectors. Regarding the origin of the PSAA and IOA, it was reported that the infraorbital artery originated in 77% of the cases from the MA and in 33% from a common trunk with the PSAA (8). The results of the present study are different from previous studies as the PSAA and the IOA had a common trunk from the MA in 60% of the cases and were separately branched from the MA in 40% of the cases. Generally, the anastomoses of the PSAA and the IOA are building a double arterial arcade in the maxillary sinus and their anatomical location is relevant in clinical dentistry.

These anatomical findings can help alleviate bleeding complications and potential injuries of the maxillary sinus arterial arcades during surgical procedures in this region. Especially in the fully dentate maxillary region, the location of IA and EA must be considered before performing any operating procedures. However, for a better understanding of the maxillary sinus blood supply and anatomy, further clinical and histological studies are needed.
